# Unraveling Reactionary Care: The Experience of Mother-Caregivers of Adults with Severe Mental Disorders in Catalonia

**DOI:** 10.1007/s11013-022-09788-z

**Published:** 2022-07-02

**Authors:** Elisa Alegre-Agís, Andrea García-Santesmases, Asun Pié-Balaguer, Àngel Martínez-Hernáez, Deborah Bekele, Nicolás Morales-Sáez, Mercedes Serrano-Miguel

**Affiliations:** 1 Medical Anthropology Research Center, Rovira i Virgili University, Campus Catalunya Avinguda Catalunya, 35, 43002 Tarragona, Catalonia Spain; 2 Department of Social Work, National Distance Education University, c/Calle Obispo Trejo, 2, 28040 Madrid, Spain; 3 Department of Psychology and Education, Open University of Catalonia, Rambla Del Poblenou, 156, 08018 Barcelona, Spain; 4 Department of Social Work, University of Barcelona, Campus de Mundet; Passeig de La Vall D’Hebron, 171, 08035 Barcelona, Spain

**Keywords:** Severe mental disorders, Institutional violence, Gender-based violence, Caregivers, Medical Anthropology

## Abstract

In most Mediterranean countries, people diagnosed with severe mental disorders (SMDs) are typically cared for by the mother, causing a significant burden on people in this family role. Based on a broader mental health participatory action and qualitative research carried out in Catalonia (Spain) of 12 in-depth interviews and 3 focus groups, this article analyses the mother-caregivers’ experience in the domestic space. The results show that patients and caregivers are engaged in a relationship of “nested dependencies”, which create social isolation. This produces the conditions of “reactionary care”, practices that limit the autonomy of those affected and that reproduce forms of disciplinary psychiatric institutions. We conclude that both institutional violence derived from economic rationality and that which stems from the gender mandate feed off each other into the domestic sphere. This research argues for placing care at the center of clinical practice and shows the need to consider the structural forces shaping it.

**Supplementary Information:**

The online version contains supplementary material available at 10.1007/s11013-022-09788-z.

## Introduction: Patriarchy, Institution, and Violence

Psychiatric reforms in advanced capitalist societies have posed new challenges for the societal inclusion of people affected by severe mental disorders (SMDs) (APA 2013) as well as for the new role of the family as the ‘de facto’ therapist (Pejlert 2001; Thompson and Doll 1982). People affected by SMDs face employment difficulties (Mangalore and Knapp 2007), their network of social relationships is diminished (Degnan et al. 2018), they suffer from marginalization, stigma, and social incomprehension (Morgan et al. 2018), their rights, including decisions about their health, are constrained (Rodriguez del Barrio et al. 2013), and they usually become dependent on their relatives (Awad and Voruganti 2008). All these aspects together form a spiral of chronicity and exclusion that limits their lifeworlds and challenges the efficacy of current mental health policies.

These challenges take on a particular shape in Mediterranean countries such as Spain, where the welfare state has historically been supported by a great moral commitment on the part of the family (Esping-Anderson 1990), with the burden that this implies for caregiving relatives, especially for mothers. In Spain (including Catalonia), the burden on mothers caring for adult children with SMDs is particularly high (Rafiyah 2011; Grandon, Jenaro, and Lemos 2008; Roick et al. 2007; Caqueo-Urizar and Gutiérrez-Maldonado 2006), affecting their physical and emotional health, their economic circumstances (Caqueo-Urizar, Gutierrez-Maldonaldo, and Miranda-Castillo 2009; Papastavrou et al. 2010), and their social relationships (Johansson et al. 2010), as well as being a source of chronic stress and intense social pressure (Ivarsson, Sidenvall, and Carlsson 2004). However, this burden has not been sufficiently addressed in the Spanish mental health care systems. It is not new, since the literature in medical anthropology (Kleinman 1988; Fabrega 1993; Kirmayer 1988; Jenkins and Barrett 2004) and in the social sciences in general have extensively shown how, in the global scenario, biomedical healthcare cultures are usually focused on mental disorders, displacing patient’s subjectivities, and their social and family worlds as issues of clinical interest (Menéndez 1984).

In the 1960s, Goffman argued that, in becoming a patient in a “total institution” such as the asylum, the affected person was deprived of subjectivity because the diagnosis occupied the self through stigma as “a special kind of relationship between attribute and stereotype” (Goffman 1963:7). Even today, and despite the psychiatric reforms, stigma occupies all the daily occurrences of the person suffering from SMDs (Jenkins and Carpenter-Song 2009; Payton and Thoits 2011; Thoits and Peggy 2015). The stigma attached to schizophrenia-related illnesses persists even in the face of objectively low levels of symptomatology, relatively high levels of social and work functioning, and a substantial subjective sense of personal improvement. It is indicative of just how hostile social response is to those perceived as mentally ill (Jenkins and Carpenter-Song 2008).

Diagnosed persons live most of their lives in the family home, have a limited social life, and tend to self-seclude in the domestic space. In this vein, the total institution phenomenon is projected into domestic spaces with inertias such as exclusion, stigma, and other forms of coercion operating in the social practices of expert and lay systems (Correa-Urquiza et al. 2006). Those affected often see their social identity reduced to the diagnosis and condition of the chronically ill, becoming “total patients” (Correa-Urquiza 2009; Martínez-Hernáez 2009), and the domestic space becoming a “total domestic institution” (Alegre-Agís 2017). Like any total institution, the total domestic institution is shaped by broader forces of structural violence.

One of these structural forces is the predominance of economic rationality in clinical practice. As Kleinman (2012) argues, what really matters is threatened by economic rationality, which shapes the options available to patients and their families as being sufficient for their experience of illness and caring. He speaks of the “deeply human” as that threshold that must not be crossed by such rationality. This paper aims to contribute to this approach by shedding light on the restrictions imposed by this type of rationality, which can turn it into a source of structural violence alongside gender-based violence.

The foundational relationship between capitalist and patriarchal rationalities, has already been raised by Federici (2004) and currently entails a mechanism of constant mutual reproduction. Thus, capitalism requires patriarchy to continue to conceal the fact that the spheres of care maintain to be assumed by someone (mostly women) without recognizing either their value or their systemic importance. In this way it is elided that “caregiving is one of the foundational moral meanings and practices in human experience everywhere: it defines human value and resists crude reduction to counting and costing” (Kleinman 2012). Paradoxically, care resists the accounting of economic rationality but at the same time requires social, cultural, and economic conditions that guarantee a fair distribution. Care makes visible the contradictions between what matters for economic rationality and what matters for caregivers and those affected. In this sense, it raises the question of whether both the lack of resources for effective care and the undervaluation of the care task can be understood as structural forms of violence.

According to Bourdieu’s *law of conservation of violence* (1999), violence is exercised every day in families, factories, workshops, prisons, even hospitals and schools, and in the last analysis, it is the product of the “inert violence” of economic structures and social mechanisms transmitted by the active violence of the people. In this sense, global, state, socioeconomic or systemic violence has an impact on individuals, who in turn reproduce violence toward others. By denying the indisputable value of structural violence in the transmission of some diseases and their stigma, agencies and social institutions contribute to fostering, sometimes through inaction, violence, and discrimination (Farmer 2004). It is important to clarify that by violence we are not only referring to visible and virulent violence, but also to more subtle, symbolic, less visible, persistent, and daily forms of it. In this respect, Zizek (2009) tells us that the subjective perception we have of the most virulent violence prevents us from seeing the violence inherent in the system in which we find ourselves. For Bourdieu, symbolic violence works in the production of domination in the intimate sphere, through the non-recognition of power structures by dominated people who collaborate in their own oppression by naturalizing the social order; for example, by naturalizing the gender mandate. Even more so because “gender is not only an attribute of bodies but runs through the blood of the institutions that end up marking the bodies and actions of those who occupy them” (Segato 2018: 65), and this, in a context of binary tradition in which to be a woman implies to be the other of the universal subject.

Following Bourdieu's argument, naturalizing the care that mothers or women in the family provide to their relatives in need can be understood as a way of contributing to the reproduction of violence and domination of these women (and of the cared for family members) by maintaining intact the conditions of unequal distribution of care. Generally, the decision to provide care is not based on a rational evaluation of costs and benefits; rather, maternal care becomes “a moral imperative” (Gilligan 1993). Maternal identity is intrinsically linked to care (Noddings 1996), and is underpinned by two essential values: the maternal instinct and maternal love (Badinter 1981). Any denial of the existence of these two elements is frequently silenced and described as sick, abnormal, or deviant. This gender mandate is internalized by mothers and interferes with their possibilities of building an identity outside the caregiver role (Phoenix, Woollett, and Lloyd 1991). In her subjectivity, the woman-mother internalizes patriarchal norms, expectations, and ways of relating in such a deep-rooted way that it is difficult for her to identify her own desires (Levinton 2000). In fact, the patriarchal mandate may coincide with the undervaluing of subjectivity and care by mental health institutions, drawing a complex scenario of structural violence.

Our hypothesis in this article is that both institutional violence derived from economic rationality and that which stems from the gender mandate feed off each other into the domestic sphere. This violence is not always explicit, but sometimes tacit, symbolic, and subtle, although no less relevant for that reason. It is embodied in coercive practices, problematic forms of care and conflictive interpersonal relationship, material actions of control and surveillance, and patterns of care that hinder dialogue, negotiation and agreement between patients and caregivers. This paper aims to unravel and understand how gender-based and institutional violence could affect the experience of caregiving in a group of mothers caring for adult children with SMDs in Catalonia. Our analysis is oriented toward the need to overcome the binary conception of care (mother-children) and the incorporation of a collective attention and networked care that considers the experiences and needs of patients and caregivers. However, before doing so, it is necessary to contextualize mental health care in Catalonia and Spain.

## The Scenario: Mental Health Care in Spain and Catalonia

In Spain, psychiatric reform stemmed from the *Ley General de Sanidad* (General Health Law) of 1986, which transferred a substantial part of the care responsibility to families (Comelles 1991). People diagnosed with SMD moved from being confined in asylums to living at home and being taken care of by their families. De-institutionalization brought a de-responsibilization of the State, which increased with the economic crisis of 2008 through cuts in the public health system. For this reason, almost forty years later, only a minority of those affected live alone, in sheltered and supervised flats, or in residential homes.

According to data from the “Headway 2023. A new roadmap in mental health” report (Bianco et al. 2021), Spain dedicates just 5% of its public healthcare budget on mental health services; a very meager percentage in comparison to Germany, which invests double that amount (11.2%). With regards to investments in (private and public) mental health personnel, Spain has one of the lowest mental health personnel-to-inhabitant ratios in Europe and the UK, ranking just above Bulgaria. In terms of quality of care and attention in services, it scores 4.3 of out 10 in social support. Likewise, in evaluations of *Individuals with poor perception of social support* and *per capita expenditure for mental health disability benefits,* it ranks last (Bianco et al. 2021).

In Catalonia, this problem is compounded. According to the Spanish Health Ministry, in 2019, Catalonia ranked second-to-last in the proportion of its GDP (Gross Domestic Product) dedicated to health (4.9%). Even more, it invested the least amount (36.6%) of its health expenditure in medical personnel (Rodriguez Blas 2019), while progressively increasing privatization of the service management; spending on private provision represented 43.6% of its total 2020 health expenditure (Accenture 2020). These figures show the decline of public healthcare services in Spain and Catalonia, and the accentuation of problems that have been dragging on since the psychiatric reform.

In previous publications (Martínez-Hernáez et al. 2020d, 2020e) that are part of the broader research that frames this article, mental health professionals in Catalonia reported specific problems, such as the lack of personnel (especially psychologists), very long waiting lists, short and closely spaced visits that do not allow a more dialogical intervention, as well as job insecurity and enormous pressure to provide care. These problems show how institutional violence does not only operate through professionals, but also on them, through the structural, economic, and working conditions in which they must develop their daily healthcare practices. The result is a drug-focused clinical approach.

In this scenario, it is not surprising that the community mental health model has not been able to be deployed adequately. The Catalan model is particularly hospital-centric and absorbs resources that are not allocated to community health (Comelles, Alegre-Agís, and Barceló i Prats 2017). While the reform was intended as deeply rooted in the community, the lack of resources allocated means that the appellation "community" has become more of an empty adjective that speaks of a *where,* rather than a working methodology, or a *how* of care. In short, the services are embedded in the community, but this does not mean that they are able to carry out a community-based social and health intervention.

Currently, the mental health care network in Catalonia has three care levels: (1) Mental Health Centers, (2) Inpatient Care Units, and (3) Psychosocial Community Rehabilitation Centers. For its part, the support system (social services) has a portfolio of financial, service, and technological benefits, such as night centers, autonomy support services in one’s own home, as well as housing services, temporary or permanent assisted living facilities, “social clubs”, pre-employment and guardianship services. However, a great difficulty still exists for labor market integration and social inclusion. This scenario leads to a situation in which most diagnosed people with SMD cohabit and are cared for by their parents. For example, in a study conducted on patients and family caregivers in Spain: 70% lived with their family; 5% cohabited part-time; the remainder (25%) did not live with the family (Vermeulen et al. 2015).

According to 2020 data from the National Institute of Statistics, 82.9% of people with a “mental disorder” were unemployed. The most common disability pension is the Non-Contributory Disability Pension (PNC), an individualized, periodic benefit for people without sufficient resources. The amount varies annually based on personal and family income, but the annual amount of an average PNC in Catalonia is 5,899.60Euros, clearly insufficient to lead an economically independent life. It also reveals the burden of care that families bear, particularly mothers. In fact, women represent 82% of caregivers, 90% of whom are mothers who undertake caregiving practices for an average of 60 h a week over 13 years, accounting for 88% of all the care received by recipients (López Gracia 2017).

## Method

This study is part of the Collaborative Management of Medication in Mental Health Project, a research and participatory action initiative developed between 2017 and 2020 in Catalonia (Martínez-Hernáez et al. 2020e). The purpose of the wider project was to apply the model of *Gestion Autonome de la Médication en Santé Mentale* (Gaining Autonomy and Medication Management, hereinafter GAM), an initiative developed in the early 1990s by research teams in collaboration with civil society in Quebec (Canada). The GAM initiative has been recognized as a good practice by the Quebec Commissioner of Health and Welfare and, since 2009, has also been implemented in Brazil through *L’Alliance Internationale de Recherche Universités - Communautés, Santé Mentale et Citoyenneté* (ARUCI-SMC) (Rodriguez del Barrio et al. 2013, 2014; Onocko-Campos et al. 2013).

The main objectives of GAM initiatives are: (1) the accessibility of information and education about treatments as a right of users; (2) the creation of spaces in mental health services to express and discuss the experience of those affected; (3) the attentive listening to the meanings and experiences of mental suffering; and (4) the promotion of meetings between mental health professionals to facilitate clinical communication (Rodriguez del Barrio et al. 2014). Our GAM project was organized in two phases: (1) qualitative research conducted between February and December 2018 (Martínez-Hernáez et al. 2020d, 2020e), and (2) subsequent participatory action during 2019 and 2020 that involved the first Guide to the Collaborative Management of Medication in Europe (Serrano-Miguel et al. 2021), and three informative audiovisual capsules (Martínez-Hernáez and Pié-Balaguer 2020a, 2020b, 2020c) on the obstacles to shared management of medication for SMDs in Catalonia.

The participants in our study were recruited from different mental healthcare services and civil associations in Barcelona and Tarragona. The inclusion criteria for recruiting patients were to be a user of public mental health services, to have a diagnosis from the schizophrenia spectrum and other psychotic disorders, and to have consumed antipsychotics for at least one year. The caregivers had to live with a patient and to be the main caregiver (not necessarily a participant), and the mental health professionals had to be in active employment. We conducted a total of 75 interviews: 38 with users (15 women and 23 men); 18 with family caregivers, consisting of 12 women (10 mother-caregivers and 2 partner-caregivers) and 6 men (4 father-caregivers and 2 partner-caregivers); and 19 with mental health professionals. Additionally, we conducted 10 focus groups, each with 6–14 participants, with patients (5 groups), caregivers (3 groups), and mental health professionals (2 groups).

In this article we focus on the experiences of the caregiving mothers, whose mean age is 65.8 years (SD: 9.51) and with a caregiving history of more than 20 years. Most of them are widowed (4), divorced (3) or married (3). In most cases the care recipient is a male child with a history of antipsychotic use ranging from 5 to 40 years and with a chronification of SMD which intensifies the care activity and makes socialization difficult, compounding the overburden on mothers as the sole figure and reference of care at present (Table 1).

The interviews and focus groups were conducted in Catalan or Spanish and at the convenience of the informants: in their homes, in public civic centers, in mental health premises, or civil mental health associations facilities. All interviews and focus groups were audio-recorded and transcribed, and subsequently coded using MAXQDA 2018 software (bi Software 2017). For the coding process, each researcher performed her/his own coding of a pre-established sample of the transcriptions (from a hermeneutic and ethnographic perspective, including the discovery of emic semantic networks) and drafted a reflexive assessment. The individual codes were agreed on through several joint meetings to determine a coding framework, discuss disparate cases, and evaluate the researchers’ reflexive accounts drafted during the fieldwork. The resulting coding tree was applied to all the interviews and focus groups, and inter-reliability was then tested by cross coding a subsample of transcriptions. In the case of caregivers, the main codes were six: communication with family and professionals; lay strategies; care experience and self-care; subjective and collective care strategies; stigma; and daily care practices. We also analyzed the four practical dimensions proposed by Tronto (2009): (1) caring about, (2) taking care of, (3) caregiving, and (4) care-receiving. That is, we considered the activity of care as a complex process that involves different phases or stages, not as a simple action initiated by the care provider.

The study applied all aspects of the COREQ criteria for qualitative interviews and focus groups (Tong, Sainsbury, and Craig et al. 2007) and was approved and monitored by the following bodies: A, B, C, and D (Anonymized). The research was carried out in accordance with the ethical standards of the Helsinki Declaration of 1964 and its subsequent amendments and was supervised by a specifically created internal body: the DoingResearchWith Committee (in Catalan: *Comité FerRecercaAmb*), composed of patients, caregivers, and health professionals, whose function was to ensure that the participants’ rights were respected throughout the research. All participants agreed to participate by signing consent forms that included an information sheet about the project’s purpose, their rights as participants, and the safeguarding of their data.

## Results: Discovering “Reactionary Care”

Our results show how care invades or absorbs the identity of mothers through three fundamental pillars: first, the essentialist and patriarchal idea of maternal care that is incorporated by mothers as a mandate for selfless care; second, the overload of care which impacts the physical and emotional health of mothers and causes them to abandon other roles in their lives; and finally, as a consequence of this situation, the surveillant care that mothers exercise against their children with SMDs through micropolitics of power in everyday life based on control and coercion. These three pillars converge in “reactionary care” (Pérez Orozco 2014), those care practices that limit the autonomy of those affected and reproduce an unequal distribution of care. Table 2 in the supplementary file includes narratives from mother-caregivers that reinforce our three-pillar model.

### “There’s Only One Mother”: The Essentialization of the Mother-Caregiver

According to the narratives of our informants, the onset of the illness imposes a temporal split in family life, generating two clearly differentiated vital moments: the time before the illness, and the time after the onset of the illness. The diagnosis is a turning point in the perception of and relationship with the child. As one mother points out:

The sad thing is that my son is not the same. He keeps doing things, but it’s not the same. The medication, just as it erases what is bad, also erases what is good. My son was very smart. I miss that.

In that sense, the formal certification of disability is experienced with ambivalence:

It is important to have the disability certificate (…) but I wish he didn’t have it and that we could burn it. The day I asked for it, I cried all the way home. When you sign it, you are acknowledging that your child has a handicap. But you must do it, he can now do a years’ study in two years instead of one, activities have been adapted to him, and so on. (Mother caregiver)

As Milliken explains, “parents grieve for a child who is gone but not dead; they grieve about the life their child now lives; and their grief is ongoing with minimal resolution” (2001:160). This social rupture—this “civil death” of which Goffman (1961) spoke—is painful for families, especially for parents. The illness entails a damage to parental expectations and, in a way, grief for the “lost child”; a kind of nostalgia for the son who could not be:

It’s a different life… We have a different child. It breaks your heart. You can no longer compare the before and after because it actually breaks your heart... it is as if that child had died. That’s another story, a previous stage. […] the child that you had and that you [have]… you have nothing, in reality, you have to learn to live [in a] totally different relationship. (Mother caregiver)

Living with children with SMDs means that “the child is permanently present” (Johansson et al. 2010:695) in the thoughts and actions of the parents. For example, one mother says “[my son] doesn’t want to leave the house with anyone [else], only with his parents or siblings”. The literature on families living with this condition provides evidence that the social organization of care represents a continuing caregiver burden and psychological distress for them and can significantly affect their functioning (Saunders 2003). Since the usual roles and kinship relationships are shifted and transformed tensions within families tends to be generated. Another mother explains: “When the others (siblings) got married, [my son’s] reaction was– “they’ve abandoned me, they’ve gone and left me”.

Saunders argues that, compared to fathers, mothers make “a greater emotional investment in caregiving roles” (2003:181). Care undertaken by women, especially by mothers, is naturalized as characteristically maternal. This is consistent with our data. As one mother states: “I always say that motherhood is quite a natural feeling, and that fatherhood is a social construct”. Thus, a gender mandate is assumed in which care is selfless and unconditional, leading to the emergence of statements such as “There are many women or men, especially women, who become complete slaves”. As indicated by our informants in one of the focus groups, the mother feels the mandate of unreserved love and non-abandonment:

Man: a mother never leaves her son.

Woman: Never. A father can leave a son. But a mother? Never.

Mothers say that, for men, it is “more difficult to accept” the damaged expectations implied by a child’s mental illness.

You go to the acute psychiatric unit, and everyone who is there during visitation hours are mothers. It doesn’t matter, you know. Divorcees, like in my case, or not, it doesn’t matter. It’s the mother who is always there. We are the caregivers, and it’s the same with your parents, you know? I have one brother and I am the caregiver of my parents. Not my brother. (Mother caregiver)

In fact, mothers internalized that “there can only be one mother” (*madre no hay más que una*), and that maternal love is unrestricted, while fathers more often abandon the family or, if they do remain in it, disengage from care tasks:

My husband just focuses on work, he tells me: ‘honey, you have your role and I have mine’. It’s me who managed the household, [...] me who took her to the psychiatrist. My husband […] maybe he asked [about her care], but it was as if he wanted to escape all that. (Mother caregiver)

A constant in the narratives of the mother-caregivers is that women should assume the main caring tasks, as indicated by the gender mandate. This means that they must abandon their own life projects or postpone them, placing their own desires, relationships, habits, and schedules on the backburner and adapting to the needs of care recipients: “Without realizing it, I noticed how things tend to revolve around [his] needs.”

The isolation experienced by diagnosed people also extends to their mothers who, even when the patients are adults, become the main source not only of care, but also of affection and socialization. Frequent in our research were comments such as “He has no friends, I’m the only one he has”, “He is very demanding with me… he is very demanding regarding what time he eats. I am getting old, and I have realized that he does not accept that I am ageing, [because] I am all he has”, or “It takes away a lot [from me] … don’t know. Not [my] liberty but, yeah, [my] independence.” The loneliness felt by the diagnosed person increases the pressure on mothers, to the extent that they have no time of their own while feeling the need to socialize and take a break from the role of caregiver. As one informant indicates:

I became a widow 2 years ago... and now I would like to go out a bit... to go out a little because we are leeches and this is not good, either. Neither for him nor myself. I would like that he had friends or whatever, to go out and talk, that he was not by himself so much. (Mother caregiver)

The situation worsens as the years go by, as caregivers increasingly spend more and more time alone with their dependent children, most especially once other children become independent. As one mother says about her and her child: *“*We couldn’t manage it, it was just us, just us.”

The mothers speak of the mental burden and the constant worry they have about their children, they suffer for not knowing what will come of them when they die, they worry about who will take care of them:

My only worry, my God, is that I get to 100 or 90 years old..., and that she dies before me. I am not worried about the economic future. My only worry is that I will die before. My only worry is if her brothers can be able to manage things for her, but they won’t go live with her, and she will be alone. If I know that I will last a whole life, I would be calmer. (Mother caregiver)

In this sense, mothers live and feel caregiving as an endless, never-ending task, even as a mission that transcends their own lives, since they not only have to unconditionally care for their children with SMD while they are alive, but also must think about and organize their care when they are no longer alive.

### “What Choice is There?” The Overloaded Care and Its Consequences

Caregivers somatize the isolation, lack of support, and overload occasioned by their experience, which frequently causes a worsening of their own physical, psychological, and emotional health. This situation is further aggravated by gender. As Cook (1988) has pointed out, emotional distress is measurably more evident in mothers than in fathers. In this regard:

Parents go too far … especially women. Why women? I speak from my own experience and from that of the other women. When our son can’t take care of himself, we treat him as a little child. If we shower every day, they have to shower every day; if they don’t do it, we get mad, and we call them filthy. We, women, act like this, we recognize it, frankly. Men care more about work, and they call them lazy. (Mother caregiver)

Our informants report numerous health problems, but they especially highlight very varied but common symptoms (i.e., depression, anxiety, chronic stress) in caregivers that are treated exclusively biomedically, as the following two narratives shows:

Mother: I feel bad, too. I don’t know if I’m also depressed because we have been like this for so long.

Interviewer: And have you ever spoken with a doctor about how you feel?

Mother: I don’t know… With my family doctor no. She knows we have a child like this.

Interviewer: And have you commented on it with anyone else?

Mother: No, no. Maybe I say something sometimes, but no, I don’t explain anything. What for?

The first day they diagnose your child, they offer you nothing, but over time as they get to know you, it is obvious that you are stressed; like, I have lost hair, I have developed alopecia. Caregivers typically end up medicated with anxiolytics, also because that’s the mantra of psychiatrists. (Mother Caregiver)

The feeling of isolation and stress leads them to even doubt their own mental health. Yet concepts such as “patience” and “stress” and the feeling that “we have to endure” were constantly encountered. Caregivers state that their diagnosed children are demanding about and rigid with their schedules; this is part of expectations regarding maternal care. Seeing that the interview lasted longer than expected, for instance, one informant repeatedly expressed anguish because her son was waiting for her to prepare food: “I have to stay in step, otherwise he gets angry, he wants everything—to have lunch, to have dinner, everything, right on the button.” Mothers strive to be constantly available in case their child needs them (Johansson et al. 2010), as the following narrative demonstrates:

I went through stressful phases. I was working in a factory, and I had to stop it because it was impossible, with the stress I was under, it was impossible. I was on medical leave for around half a year, for my back, but the doctor told me- ‘This is due to excessive stress that goes straight to your back’. And he said, ‘if you continue like this, you’re going to end up in a wheelchair’. The whole family went through a really bad process, because they are tyrants. Careful though! When you have an ill person in the house, whatever it may be, it doesn’t matter who you have on your side helping, what you have to endure is a lot because normally the ill become a tyrant. Yeah? (Mother caregiver)

The most trivial aspects of everyday life must be adapted to the needs of the patient, which creates a domestic climate of stress and anxiety. In this setting, the possibility of a violent reaction inhibits certain patterns of relationships and socialization:

Mother: He insults us; indeed, he does, he treats us... well, he just screams straight away. Straight off he’s very aggressive... and you can’t contradict him or say anything to him because it just totally riles him.

Interviewer: And how do you handle this? What do you do?

Mother: We come home and turn on the television, but he tells you that it is the devil’s channel and turns it off... He takes the remote control, switches the channel, and turns off the sound. And that’s how it is. All day long.

Mothers feel they are expected to take on the care burden of persons with SMDs without sufficient professional support or social recognition. This situation is aggravated by the lack of information on the health status of patients, especially at the outset of the psychiatric trajectory, mainly due to data confidentiality and patient rights issues. As one mother says: “I think I should have a minimum of contact with the psychiatrist because I live with the patient.” But despite the concern and the need to know, the usual response is “I’m sorry, I can’t give you that information.” It is taken for granted that the family will follow the therapeutic indications even without being informed about them and with no recognition of their fundamental role, as one mother argues, the caregivers should have “all” the information about their children:

Those of us that live with them should know everything... my son at first tore up all the [medical] reports. And if you asked for a duplicate [from the psychiatrist] they wouldn’t give it to you. This is a big problem because it’s the family who lives with the ill person who really knows [him/her]. The caregiver is the one that, in reality, should have the right to know the ins-and-outs in order to know how to react. There isn’t a family-doctor communication. There isn’t any.

Family caregivers want and need more information and respect from mental health professionals. Usually, they feel they do not receive the same recognition as caregivers of people with other diseases (Winefield et al. 1998). Mothers mostly understand SMDs as a “disease” and, consequently, their children as sick and dependent:

I had an argument with the head of the adult mental health center. I told him, ‘look, I am his mother, he is living with me.’ If I have a child or a partner or a family member who has cancer or liver disease or kidney disease, the doctor will tell me what treatments I have to administer, how I have to take care of them and what I have to do. And with this person who is not able to function in their own right because of a mental disorder, it turns out that you are under the obligation to not give me any kind of information. Well, you know, that seems horrible to me. (Mother caregiver)

Two parent caregivers explain that they are not allowed to talk to their child’s psychiatrist and that the therapeutic group for relatives no longer exists. Thus, they feel abandoned because of the lack of support and information from the public mental health network. This is seen as unfair, since the caregiver role is not assumed by choice, but is often regarded as the only option in the absence of public resources: “we don’t have a system that frees us from that caregiver role, many of us do this simply because there is no other option”, says a mother. The lack of information also has an impact at the symbolic level because it implies an underappreciation of the role of the caregiver while they acquire knowledge and experience, and this often makes them feel that they are experts. However, the dissonance between their lay knowledge, compared to expert knowledge, and the lack of recognition—one of the reasons for their unease and confusion—highlights the power relations that relegate, undermine, and devalue the knowledge of mothers. As some of them indicate:

It surprised me. In fact, that’s why I and my son went back to the previous one. The psychiatrist didn’t ask me how I saw him in those two months that he had not seen him, because this is a disorder with symptoms, not one in which physical tests check the level of schizophrenia. So, if they do not ask me, not necessarily when my son is present, but on the side... (Mother caregiver)

I have no rights. I had to go and ask if my son was keeping his appointments with her (the psychiatrist) or not, and when she looked at the computer, she said: ‘I’m sorry, but I can’t tell you’, and I said, ‘I’m only asking you to say yes or no’, and finally one day she said, ‘look, calm down, relax’ and from there I deduced that he was going to the appointments. But we went to talk to the social worker and [they said] ‘no, no, he rejects everything, we have proposed this, we have suggested he goes to the psychologist, we have suggested a center. He rejects everything’. (Mother caregiver)

This conflict is due to the coexistence of two contradictory principles within the mental health network regarding SMDs: the biomedical principle (people are diagnosed as sick and dependent), and the citizenship principle (diagnosed people are also subjects of rights). In fact, the mothers' demands reflect the lack of capacity of mental health care services to resolve this controversy, largely due to the lack of human resources. Mothers are thus caught in a structural conflict. On the one hand, they are expected to take care of their children's lives. On the other hand, and in order to safeguard the rights of those affected, they receive scarce professional information on the evolution of the disease.

### “Please, Take the Pill!”: The Surveillant Care

Most caregivers point out that the worst moment occurs at the beginning of the psychiatric trajectory of their affected children, with the first psychotic episode, especially if caregivers perceive the impossibility of dialoguing and negotiating with their children. At that moment, caregivers recognize that they defensively tend to reinforce control and coercive mechanisms. If individuals with SMDs become violent, family members are most often the victims, especially mothers (Copeland and Heilemann 2011). There are even extreme cases in which violence is physical and pervaded by gender-based violence, as in the following case:

It got to the point that he sexually assaulted me. He wouldn’t let me pass [by him], and it gave me this impression, until he touched my breast. He said: ‘mama, it’s because I’m crazy.’ They gave him a ton of things, and nothing worked. On other occasions he became aggressive, later throwing the medication. He was admitted by judicial order, twice. Calling the police so that they could come and look for him… (Mother caregiver)

Potential aggressions lead to mothers living in a constant state of alertness, uncertainty, and even fear. The situation of tension is aggravated further if, as is often the case, the father figure is absent. Another frequent issue is that the care recipient, due to his/her mental diagnoses, distrusts family members, and accuses them of persecution, abandonment, abuse, or even attempted murder, as a mother describes: “Yes, he began to see strange things, that someone was poisoning his food… many things.” In fact, there are mutual fears, which lead to a confrontational logic of “it's either you or me”, whereby both parties can become perpetrators and receivers of violence.

This situation generates emotional distance and mutual distrust, becoming extreme in cases where families must turn to institutions to try and restrain the patient. Hospitalization is almost always understood and experienced as a threat by those affected; it is rarely seen as a support, but rather as a punishment. Previous negative or traumatic experiences in the psychiatric circuit contribute to a lack of trust in the family in the face of threats of hospitalization. At the same time, although parents consider they are doing the best they can, their feeling of guilt is constant (Peljert 2001).

Stress and the lack of resources to support families favor involuntary admissions or the threat of their occurrence. This leads to problematic forms of care characterized by confrontational relationships between care recipients and caregivers. In situations of burdens and stress entails abandonment that easily drifts into various forms of violence:

He is enraged. Enraged. He insults us. Sometimes it makes you want to punch him the way he behaves with you and insults you. But I do not hit him because if I do, it’s me who is screwed if I’m reported. But that’s how it is. There are times like that. [We feel] rage. So, you say, ‘well, just see how you treat us, young fellow’. (Mother caregiver)

Over the years the patients usually learn to dialogue and agree on shared solutions but according to the informants, this is only possible to the extent that there is disease awareness. As a mother explains: “They must accept they are ill. If they accept that, we can talk about anything. And if you accept that too, you also accept everything that comes with that.” This question is problematic and is related to the imposition of a certain interpretative framework on suffering. The awareness of disease, which is rejected by many patients, often clashes with the awareness of suffering (Martínez-Hernáez et al. 2020e), which does not necessarily entail the acceptance of specific clinical category such as schizophrenia or the consideration of sick person. The appeal to awareness of disease is related to adherence to treatment and is the only way to be cared for in the mental health network. In fact, here the mother is expressing this unique pathway to care that connects with the existing care resources.

Mothers are aware of the undesirable side effect of drugs (Martínez-Hernáez et al. 2020e), but the widespread perception is that “meds have many side effects, but they have more benefits than secondary effects.” Feeling trapped and persecuted, families often have little choice but to use coercive and monitoring mechanisms, given the lack of other resources (Milliken 2001). Certain synergies of demands, in the rigid domestic space, are embodied in the figure of the care recipient who understands that these are the mother’s ‘obligations’, while the mother also understands and incorporates them as such. For example, basic, everyday tasks become a scenario of control and supervision of the children by the mother: “(It’s) a constant vigilance, there’s no rest. And they don't do much, but they know that they have to take their medication, make their bed, take a shower… eat…” (Mother caregiver).

The human cost is mutual enchainment and “nested dependencies” (Kittay 1999) between the caregiver and the care recipient, along with control, surveillance, and coercion. In this sense, some mothers say: “He doesn’t want to take the medication. I have to spy on him” or “sure, the doctor knows I'm monitoring. He says, ‘the mother has eyes on all side of her head’” or “Now he doesn’t want to take the pill. So, the doctor knows and won’t change it. Depending on it goes, I will speak with the doctors, without him [present].”

When the main caregivers (in their isolation) feel ground down by the pressures of care, they also feel authorized to impose (also in their isolation) the necessary norms and mechanisms to cope with their situation. While family control could be more or less extensive, it usually takes its ultimate expression in medication management:

Once he said, ‘I don’t want to take the pills anymore’, quite aggressively, and when he said that he didn’t plan to take the pills. I went into his room, packed a bag for him – he was 19 years old – and told him that he could leave. I calmly said, ‘If you decide not to take the pills, you leave my house because I do not want to go through this all over again.’. He saw me so convinced that, well, he unpacked his bag, took the pills and never said anything again. […] The message that I transmitted was that you have to take this medication, just as others have to take things for the heart or whatever. It’s an[expletive] but that’s how it is. Accept it. (Mother caregiver)

Part of the reasons underpinning this supervisory and control role are to be found in the wear-and-tear experienced by caregivers, given that “caretaking mothers have been thrust into a position where they are expected to provide care to their children, without support and at times under the threat of impending physical harm” (Copeland and Heilemann 2008:12). In fact, imposing a routine is a form of caregiver self-care, as they avoid having to deal with the same situation repeatedly. As a mother expresses: “When one has or has had many battles … for example the battle to get him up and out to work every day … Well, when it comes to preparing the medication, there is no battle. I just go and prepare it.”

## Discussion and Conclusions

This study describes how gender mandate and institutional violence operate in the ways that care is given in the domestic space. We have been particularly interested in understanding the impact of this violence on mothers of adult children with SMD, and the incidence of this violence in the production of coercive forms of care. The economic rationality of mental health services together with the gender mandate find themselves in a problematic scenario for caregiving. The lack of recognition of the importance of care (Kleinman 2012) leads to the neglect of caregivers and consequently to strain and lack of support.

Patients and caregivers are involved in a relationship of nested dependencies (Kittay 1999).

We use this concept to highlight a problematic situation of obligation and imposition between the caregiver and the cared for, but not to support the Kittay’s idea of resolving this circumstance by the sole means of satisfying the caregiver’s care needs. In our view, the dual relationship between the two parties and the responsibility that it entails must be opened-up to other actors and consequently to collective care. Here we align ourselves with Back (2015) in calling for a broad social response to situations of dependence, co-dependence, and inter-dependence. All of them are inherent to the human condition and not exclusive to some groups. The moral demands of caregiving, intersected with the female gender mandate, in a context of inequality, positions caregivers in a situation of fragility and problematic dependency. However, the problematic focus is on the social conditions for care and not exclusively on the individual material conditions of caregivers. In other words, caregivers become dependent not because they take care of another dependent person but because the conditions under which they take care of another dependent person are inadequate and they have consequential nested obligations.

Furthermore, an inadequate accompaniment by mental health services produces “reactionary care” (Pérez Orozco 2014), practices that limit the autonomy of those affected, and are the fruit of a structural situation of an unequal distribution of care that is aligned with capitalist economic rationality. These reproduce disciplinary forms, typical of the total institution, in the domestic sphere.

On the one hand, diagnosed people feel (and are) socially isolated due to a process of marginalization, lack of social resources, and overmedication. Caregiving mothers, on the other hand, also feel isolated, due to the overload of care and the lack of sufficient professional support. The care routines, the affected web of dependencies, the feelings of guilt, and the burdens all have an impact on their emotional and physical health. This daily suffering is silenced, assumed as part of the self-sacrifice prescribed by the gender mandate. The idealized view of maternal care (Gilligan 1993) shows its contradictory face in our ethnographic findings, where the unbearable burden of motherhood becomes an everyday affliction.

Mothers must deal with the ambivalence that defines the expectations surrounding their role: they must promote and respect the patient's autonomy, and at the same time, they are responsible for their medication, behavior and well-being, and must therefore monitor them. In this context, the care burden is often resolved by the mother-caregivers taking power and control, as a way of circumventing conflicts, economically managing time, and even re-appropriating their own life. The gender mandate of care is not just a question of a nice ethic or of unpaid philanthropic work, but also a form of coercion that produces violence in multiple directions and in which caregivers and care recipients are caught in a loop of social needs and constraints (Pérez Orozco 2014). Therefore, as in other situations of violence, it is important not to confine domestic care exclusively to the realm of intimacy (Segato 2018) as it is also a structural and systemic product.

Violence is part of the daily life of mother-caregivers who, while suffering psychological, verbal, and even physical attacks, at the same time exercise control, surveillance, and coercion over the care recipients. “Surveillant care” grants the mother-caregiver power which is sometimes invisible and underappreciated in family relations. In many cases, this power is lost in other areas of the women’s lives, because they have had to forsake careers, social relationships, and leisure. Our analysis does not point to the mothers as “guilty”; on the contrary: we want to highlight the consequences of an unequal distribution of the care burden. Care involves a potential for coercion and violence, and denial of this dimension does not solve the problem nor minimize its impact. To prevent it, violence must be recognized as a possibility.

Ours is a critique of the structural social conditions that generate both an unequal care system based on the unfair distribution of time and tasks (gender-based violence), and the undervaluation of the social function of care (economic rationality violence) (Hughes et al. 2005). Adequate community mental health care cannot ignore the social context of care and the household production of mental health and care. The social suffering involved in caregiving must be considered in mental health policies. In our study, mother-caregivers feel their voice disregarded by expert mental health systems, for example when they are excluded from decision making and professionals do not inform them about their children's health status. We agree with Milliken and Northcott that “professional caregivers must acknowledge the contribution that families make to the patient's therapy, but [must] also accept professional responsibility for providing care to family caregivers” (2003:111).

In conclusion, care cannot and should not be considered in binary giver-recipient terms, but as a common project where responsibilities are shared and should (perhaps) be defined and agreed on in advance between all the parties. Like Stone (2019), we support the idea of entangled care and of porous boundaries between care system, caregiver, and care recipient to overcome the traditional fiction of binarism: care is not given in advance but is constructed and evaluated in daily negotiations (Winance 2010). Our research points to the need to place care at the center of clinical practice, and to organize it in a network of reconfigured burdens and responsibilities.

Some of the mothers who participated in this research co-authored with users, health professionals and the researchers of this project the Collaborative Medication Management in Mental Health Guide, a tool to facilitate shared decision making and cooperation between users, and professional and lay care networks. As in the previous GAM experiences in Canada and Brazil, the aim of this guide (Serrano Miguel et al. 2021) is to promote a more equitable distribution of responsibilities and the articulation of a support network capable of attending to the patients as well as the rest of care actors in Catalonia. For example, one of the objectives is relieving the care pressure on mental health professionals. This can be done by activating possible community actors who are currently not considered relevant even though they are significant for users. We believe that this networking collective work can resolve part of the conflicts between the right of family members to be informed and the right to privacy of patients. The former being formally incorporated into the collective work of care or, on the contrary, by activating support networks for patients that will reduce the responsibility and burden on families. In short, it is a matter of moving away from the logic of confrontational rights between relatives and patients toward a healthcare culture based on ethics of care. This is likely to be one of the most urgent mental health policy challenges facing care for people with SMDs and their families in the coming years.

**Table 1:** Detailed information on mother-caregivers and their children

Interview	Mother age	Civil status	Co-habiting	Son/daughter	Age	Years of medication
FM.06	67	Divorced	Mother and son	Son	47	30
FM.02	60	Married	Mother, father and son	Son	40	20
FM.03	84	Widow	Mother and son	Son	60	40
FM.04	45	Divorced	Mother and son	Son	20	5
FM.09	68	Widow	Mother and son	Son	45	20
FM.10	60	Widow	Mother and son	Son	41	20
FM.11	60	Married	Mother, father and son	Son	40	18
FM.16	62	Divorced	Mother and son	Son	36	16
FM.08	67	Widow	Mother and daughter	Daughter	47	30
FX.07 (Couple Interview)	80 (w) 82 (m)	Married	Mother, father and son	Son	47	30

## Supplementary Information

Below is the link to the electronic supplementary material.

Supplementary file1 (DOCX 44 kb)
