# Supplementary material for: Unraveling Reactionary Care: The Experience of Mother-Caregivers of Adults with Severe Mental Disorders in Catalonia
Source: Cult Med Psychiatry. 2022 Jul 2;47(3):790–813. doi: 10.1007/s11013-022-09788-z (PMC10406675; doi:10.1007/s11013-022-09788-z)
Supplement: Supplementary file 1 — Supplementary file1 (DOCX 44 kb) [file 11013_2022_9788_MOESM1_ESM.docx]

**Supplementary data. Table 2: narratives of mother caregivers**

| Essentialization of mother, caregiver’s burden, and changes in patient’s life | Reactionary care, surveillance, and institutionalization of care | Problematic Relation with professionals and health care services |
| --- | --- | --- |
| There is an underground machismo in the world of psychiatry. They told me: "maybe it's your perception." *(mother)*  The last time I had to accompany him, although he never likes me going to the doctor [with him], to negotiate with the doctor, because he must think: ‘if my mother goes, she will negotiate and she knows how to refute the doctor, whereas I don’t know how to do that. I don’t have enough authority, so if my mom goes, things are different...’ and it was like that. *(mother)*  [I tried] to break the umbilical cord with him. We have breakfast together every day because he’s always buzzing at my door [calling me]: “mama”. He doesn’t have friends; I am his only relation… *(mother)*  It’s better if the person gets better. You can’t have your child living like the living-dead [from the medications] in their room. (*mother)*  If there is just one psychiatrist who says you are schizophrenic, your rights have ended. *(mother)*  They don't have anything to do. They don't have a life of their own. They don't have money... well, they end up in bed [all day]. *(mother)*  What it [the medication] does is pacifies the subject. It is impossible to manage anything. We have turned him into an object, not a subject. *(mother)*  Taking so much medication everything has been lost. He couldn't get up to go to university. *(mother)*  After the breakdown my son was... Forget about what he was before... You can’t even compare it because there’s no comparison. He still has some things [from before] that yes, when he is stable yes, it’s him. But realistically he’s never the same. No, no... And they come back less trusting. They are almost a bit autistic and go... Nothing in comparison. Absolutely nothing. *(mother*)  [The medications] completely remove their personality. It cancels them. *(mother)*  My son was very intelligent. I miss this very much. The medication has erased him. It has erased the bad and the good. *(mother)*  I can tell you is that it [the illness] definitely starts to isolate them from their social surroundings, above all when they are diagnosed at a young age, like my son [was] *(mother)*  He tells everything. He tells me everything. He is very infantile. Because he started so young... He is like a little kid. (*mother*)  What happens is that I must follow his rhythms, otherwise he gets angry, he wants everything: food, dinner, everything on time and on schedule ... [it takes] a lot of patience. (*mother)*  He doesn't do anything at home … the computer, the bed, and the television. And messes things up; puts mountains of things on the couch, the table. I scold him, to have a little autonomy. Use it for something! (*mother*)  The worse will be when I’m not longer... His father is dead. But when I’m not around... Let’s see if God wants, if more things will be available, like shared housing or whatever. As he doesn’t speak much, doesn’t have many friends... If he was in a shared flat, [with] maybe 4 or 5 [others], he would do his own thing. (*mother*)  The patient’s opportunities should not depend on the family. Things need to be done [by the health services] to ensure that they do not lose skills. (*mother)* | I heard him at night that he was not sleeping ... because I know when he is not sleeping. When he is sleeping, he has breaths normally and calmly… and I hear it in the hall. I tell the doctor, and he says: ‘Sure, if you didn't look at him so much …' The doctor also scolds me. (*mother*)  Some sons or daughters live alone and [the parents] bring them food. I would suffer a lot. Not having him at home, I would be suffering more, we would be calmer, yes, but we would suffer. There was a mother who couldn't see [her son] and [you] can't know what he does ... I have him at home. I control everything, I control the phone, I control the medicine, and more, right? (f*ather*)  I disinherit him. That is automatic. If he tells me to stop (the medication) I disown him. (*father*)  I will not support his demand to not take the medication (without the consent of the psychiatrist) because he will lose his mind. *(mother)*  When they are in crisis there is no possible negotiation. There is no negotiation. I think it must be imposed. But once he’s stabilized and back to normal, then I think that he would have to be talked about. (*mother*)  If a person is in a crisis, you cannot negotiate anything with her, they must take the medication and that’s that. (*mother*)  I think they are a kind of sick people who, if, for example, they are asking for something, for example, to reduce the medication or give them another one. If they don’t feel listened to, they get nervous, they get angry, they get upset. In the end they will be upset, they will be angry because “hey, this medicine is going badly for me and they’re insisting that I take it”. They get obsessed with it. In the end, they are worse than if you reduce the meds a little bit or change it for another. (*mother*)  He is in bed all day, if we take a long time to come, he calls us because he misses us, but when we arrive, he bothers us. It's a daily struggle, it's a daily abuse. *(mother)*  He thinks I want him in the hospital, and I want to get rid of him. He fought with everyone. He became very violent with his children, and they had to hospitalize him. He didn't go voluntarily. We had to call the police, the ambulance came, and for him that has been quite grave, and he says it's me, and he doesn't trust me. I don't think he trusts me. (*mother*)  We could never control her. She has lived with me, and we have endured the crises and aggressions. (*mother*)  *Interviewer:* And how do you normally act in these situations? When he wants to stop taking or reduce a medication?  *Mother:* Well look, I explain it to his doctor and if not, we call and we push the appointment forward.  *Interviewer:* You don’t intervene?  *Mother:* No, no because he would not pay me any mind and would do the complete opposite. No. No. I have already learned that, that this is his doctor’s [issue] because if I begin to argue with him and we begin to argue... Lately, I do not argue with him at all, he always gets angry. [I say:] “Hey. No problem. Listen, you speak with your doctor as he’s the one who understands all of this. I don’t understand anything.” (*mother)*  The ones they love the most are the ones they treat badly. My son adored me and accompanied me. Now he doesn't want to accompany me, not even to the corner. I explained to the doctor, “now my son is against me. He`s neurotic.” So, they could medicate him. And as he [my son] pays us no mind, well, he suffers. He tells me, “Mama don’t get angry”, and I reply “no, I’m not angry, but come with me...” [then he says] “no, no I won’t come with you.” *(mother)*  He wants everything when he says so, and if not, he gets angry. I have to have a lot of patience. It’s his character. He only has me. When he has a doctor’s appointment, he doesn’t want me to go with him, because he doesn’t want me to explain certain things because he gets angry. But he’s not as violent as he used to be. *(mother)*  I built him a separate house on our property. He’s loving his life in his house. We can’t be under the same roof because things will end badly. That I’m sure of. *(mother)*  He takes a lot away from me. Not [my] freedom but, yeah, [my] independence. *(mother)*  Medication is a medical issue, as the medical word is the doctor’s word. The family should support the doctor, and say, “hey, he has studied, and you do what he says, because it’s the same as the any other disease.” I think the role of the family is to reinforce the doctor’s decision. The doctor should try to explain to the patient the benefits s/he will have with the medication and the effects, or not, it will have. (*mother*)  In my experience, I think the worst thing is to contradict him. And that’s one of the few things I’ve learned: when they become angry or are determined [to do something] do not contradict them. It’s that they get more upset. They get upset... I’ve seen them do it to the professionals. Or if a professional [contradicts them], it ends up in an argument. The next day they come to their sense and if you agree with them a bit, they come back [themselves]. Now, when you openly confront something “Don’t. No don’t. No. Don’t...” well, you can even have a crisis. (*mother*)  For example, he told me “Dr. X told me to take [medication] to sleep, and if I don’t take it? And if I change it for something else?” I always tell him “No.” I can’t tell him something different to what the doctor has told him, because the next time he will stop taking or doing something. I cannot interfere either with the doctor, when he asks to reduce or increase. I always tell [my son] to talk first with the doctor about this. That’s why the doctor is there. (*mother*)  He lived in the streets until he relapsed, and we reported him to the judge. Usually, we were lucky because the police always caught him beforehand, so the police would call us. I always told them: “No. Take him yourselves because if I go, he will get very nervous. Try to take him. I’ll head to the hospital.” And that was it. They tried. Sometimes they managed, and sometimes they didn’t. So, of course, I said: “well, I’m not moving from here because my son, yes, I know him, and I know he is ready to be admitted. I am very sorry, but I am not taking him home. (*mother*)  An involuntary admission and that’s it. But it’s hard, it’s hard... Not everyone can do it. It’s very hard, because you have to report your own son! Look, it’s difficult. And then, if the partner doesn’t completely agree, that’s another thing... and there are a lot of couples that break up. (*mother*)  *Interviewer*: And if he suggests stopping or reducing the medication, would you support him?  *Mother*: I would support him as long was a knowledgeable person was with him, or that he was in a place where they were controlling him, not me being in control. Because if you leave him alone, he commits suicide. I’m scared to find him dead, if he commits suicide in my house. (*mother*)  I have mothers and friends obsessed with... [they say] “Don’t stop taking the pill” ... “Injections are better, because this way it’s not forgotten.” The fear. The fear that psychiatrists instill in us about stopping the medication, makes us complicit in this perverse situation. However, this does not help them to be empowered, nor does it help them to learn to manage their differences, their difficulties... (*mother*) | We need training for families, throughout the process. That the psychiatrists explain to us, and that we could explain the day-to-day symptoms to them. But they don't teach us anything. Families need to know everything… (*mother*)  They closed the asylums and didn't give you an instruction book. They are no longer locked up there, which I find horrible how they were treated. But they have sent them with the family, and let the family take care of everything, without any explanation. (*mother*)  I think it is essential that families inform us, not wait for them to inform us. I wish the system was as it should be, but we don't have it. We must live with what we have. (*mother*)  They [the professionals] don’t give me many explanations because they say it’s a very personal thing. They don’t explain to the family how he is. (*mother*)  What do they explain to us? That he suffers from paranoid schizophrenia and that he will have to be on medications for life. But we mustn’t worry because he will be able to lead a normal life if he sticks to the treatment and is aware of the illness. These are the psychiatrists’ mantras, these two sentences. But I see my son is going to get worse; also, cognitively worse. (*mother*)  Families are very lonely .... These are diseases that affect all the dynamics of the house. I turn to my son's needs. The family must depend on their common sense; you are very much left to your fate. (*mother*)  *Interviewer*: How do you think accompaniment can be improved?  *Mother*: By communication. By calling him every day, “how are we doing?” It would have to be the nurse, not me. I am the mother. I am the concerned party, and it is like a very exhaustive control, because they know, in fact, that you know them very well. It would have to be a person with more power, who is an unknown person to him. That [person] would be a nurse that has a certain trustworthy. Not the involved parties, like the father or mother. (*mother*)  They told me: if your son does not want to come, we cannot do anything. He has to be aware of this. But I said, “if he is not aware of [his] illness, how is he going to be aware? (*mother*)  They did not give me any information and as he does not allow me to go to the doctor. So, no, I cannot ask him. (*mother*)  A young psychiatrist presented the possibility of electroshock and I had read about it and how it’s included in a psychiatric therapy. It’s mind-bending. I rejected it. I said ‘no’, but then the psychiatrist turned to look at his father, to get his opinion. And then, it was me who looked straight at her and said, “don’t look for the acceptance from his father...”. I said, “because, even if he wanted, I would not allow it. He (the son) lives with me. His father has a voice, but not a vote in this matter because the first one to get screwed up is going to be my son, then me. I am the one who takes care of him. Then the doctor said to me, “well, do you believe that you know more than me?” and I said, “I don’t have the degree, ok?! Not like yours. But maybe I have read more than you, and above all, I see my son every day and I see how he has evolved, so: it’s all for nothing.” (*mother*)  He went to a day center. Now he no longer goes. When he went to the day center, he’d wake up at nine in the morning and would have an activity for an hour and would walk back which is already an exercise itself. Now, he has activities only two days a week. The other days he does not wake up until one (pm). Sure, there aren’t enough resources. The issue is that there are so many [of patients], there are only so many places ... so they split them [days of activities] up. (*mother*)  Everything is focused on the fact that they are all chronically ill. In the early years, it’s focused on the care rather than trying to fight for normality in the early years. My son wanted to do sports and they [psychiatrists] told me that I was going to frustrate my son. You cannot have an 18yo in a care plan, with no expectations. It’s all very welfare based, the (therapeutic) groups are very good, but there have to have other options. I know I have to speak more positively [at home]. Another problem is the lack of resources for some families. It is not fair that everyone depends on the family. (*mother*)  Another problem is the few hours they are attended to. (*mother*)  Although he can call when he needs, he does it on his own initiative. He only gets visits every 4 months. That’s too long [of a wait time between visits]. (*mother*) |
